# A Cross-Platform Comparison of Affymetrix and Agilent Microarrays Reveals Discordant miRNA Expression in Lung Tumors of c-Raf Transgenic Mice

**DOI:** 10.1371/journal.pone.0078870

**Published:** 2013-11-12

**Authors:** Valerio Del Vescovo, Tatiana Meier, Alberto Inga, Michela Alessandra Denti, Juergen Borlak

**Affiliations:** 1 Centre of Integrative Biology (CIBIO), University of Trento, Trento, Italy; 2 Centre for Pharmacology and Toxicology, Hannover Medical School, Hannover, Germany; Ajou University, Republic of Korea

## Abstract

Non-coding RNAs play major roles in the translational control of gene expression. In order to identify disease-associated miRNAs in precursor lesions of lung cancer, RNA extracts from lungs of either c-Raf transgenic or wild-type (WT) mice were hybridized to the Agilent and Affymetrix miRNA microarray platforms, respectively. This resulted in the detection of a range of miRNAs varying between 111 and 267, depending on the presence or absence of the transgene, on the gender, and on the platform used. Importantly, when the two platforms were compared, only 11–16% of the 586 overlapping genes were commonly detected. With the Agilent microarray, seven miRNAs were identified as significantly regulated, of which three were selectively up-regulated in male transgenic mice. Much to our surprise, when the same samples were analyzed with the Affymetrix platform, only two miRNAs were identified as significantly regulated. Quantitative PCR performed with lung RNA extracts from WT and transgenic mice confirmed only partially the differential expression of significant regulated miRNAs and established that the Agilent platform failed to detect miR-433. Finally, bioinformatic analyses predicted a total of 152 mouse genes as targets of the regulated miRNAs of which 4 and 11 genes were significantly regulated at the mRNA level, respectively in laser micro-dissected lung dysplasia and lung adenocarcinomas of c-Raf transgenic mice. Furthermore, for many of the predicted mouse target genes expression of the coded protein was also repressed in human lung cancer when the publically available database of the Human Protein Atlas was analyzed, thus supporting the clinical significance of our findings. In conclusion, a significant difference in a cross-platform comparison was observed that will have important implications for research into miRNAs.

## Introduction

MicroRNAs [miRNAs) have become the subject of intense research in recent years. These small RNA molecules are a large class of 18–25 nt-long non-coding RNAs and act as negative regulators in the translational control of gene expression [1]. By base-pairing miRNAs interact with the 3′UTR of target mRNAs to facilitate the recruitment of a ribonucleoprotein complex that either blocks cap-dependent translation or triggers target mRNA deadenylation and degradation [2]. Around 30% of human genes contain in their 3′UTRs seeding sequences for one or more miRNAs [3].

There is overwhelming evidence for miRNAs to play decisive roles in most biological processes and these include development and morphogenesis [4], [5], cell proliferation [6] and apoptosis [7]. Aberrant activity of one or more miRNAs have major implications in disease onset and progression and miRNAs were reported to be abnormally expressed in human malignancies, including leukaemia [8], breast [9], lung [10] and thyroid cancers [11]. Thus, miRNA expression profiling is of great interest in biomedical research and can be used to successfully classify stage, subtype and prognosis of cancers [12], [13]. It has been applied to cell lines, tissue and tumor samples, from fresh, frozen or FFPE (Formaline fixed paraffin embedded) materials for an estimation of the quantitative change in the expression of miRNAs populations and their regulation amongst different biological conditions or between different drug treatments.

A range of methods have been used for the simultaneous expression analysis of hundreds of miRNAs, based on microarrays, quantitative reverse transcription PCR (qRT-PCR) and next generation sequencing (NGS) techniques [14]. These methods face several challenges, due to unique features of miRNAs, notably 1) mature miRNAs are very short; 2) miRNAs belonging to the same family can be very similar in sequences, varying as little as one nucleotide; 3) miRNAs may have several isoforms, due to single nucleotide and length polymorphisms, and due to RNA editing. In particular, these characteristics of miRNAs represent hurdles that need to be overcome in the design and in the hybridization of probes or primers for microarrays and qRT-PCR techniques, respectively [14].

Several commercial microarray platforms are available for miRNA detection and quantification, and their performances in measuring the differential expression of miRNAs have been evaluated in a number of papers, often comparing them with qRT-PCR and/or NGS techniques [15]–[26]. Most studies have demonstrated differences and biases between various microarray platforms in their ability to determine miRNA expression profiles.

Differences between platforms can be due to technical procedures such as the enzymatic reactions and amplification steps performed in the preparation of the RNA samples, or may stem from microarray probe design, microarray manufacturing, detection hardware, or algorithms for the extraction of intensity signals and subsequent data analyses.

A direct comparison between the Affymetrix and Agilent platform for miRNA quantification has been performed only recently [20], [21], [25], [26]; however, the results are conflicting. Furthermore, in such inter- and intra-platform comparisons an identification of differentially expressed miRNAs was not attempted to define disease-associated regulations.

In this study we analyzed the expression of miRNAs in the lungs of SP-C/c-raf transgenic mice. The transgene is under the transcriptional control of the surfactant protein C (SP-C) promoter and consists of the oncogenically activated NH2-terminal deletion mutant c-Raf-1-BxB. This transgenic disease model develops lung adenocarcinoma that is preceded by localized proliferation of atypical cells lining the septi of alveola, i.e. atypical adenomatous hyperplasia. The molecular characterization of lung dysplasia and the cancer genomics induced by c-Raf hyperactivity was the subject of two independent reports [27], [28].

Notably, the proteins of the RAF family (a-raf, b-raf and c-raf) are serine/threonine kinases and are related to retroviral oncogenes causing cellular transformation. The Raf proteins are one of the best characterized Ras effectors activating the mitogen-activated protein kinase (MAPK) signaling pathway [29]. RAF directly phosphorylates and activates MEK via two conserved serine residues in the kinase activation loop of MEK [30]. Activated MEK then directly phosphorylates a conserved tyrosine and threonine residue in the kinase activation loop of ERK [31]. The MAPK pathway is deregulated in many human malignancies through aberrant signaling upstream of the protein and by activating mutations of the protein itself, both of which induce a proliferative advantage. Mutations of the K-ras gene have been identified in up to 30% of lung adenocarcinomas and have been considered as a poor prognostic factor [32] highlighting the important role of this pathway in human lung cancer.

We previously reported the molecular characterization of lung dysplasia induced by c-Raf-1 [27] and in a subsequent study described the regulatory gene networks involved in the transition from dysplasia to advanced lung adenocarcinomas [28]. We also explored the tissue and serum proteome of lung adenocarcinomas in these animals [33], [34] and now extend our analysis to an identification of regulated miRNAs in cancer gene networks. Our study focused particularly on precursor lesions of lung adenocarcinomas, and we compared data obtained by the Affymetrix and Agilent platforms and by qRT-PCR.

Overall, this study focused on an inter platform comparison by assessing the reproducibility of data obtained and secondly investigated the functional importance of differentially expressed miRNAs in precursor lesions of lung cancer.

## Results

### miRNA Profiling Using Two Different Microarrays Platforms

Two microarray platforms for the analysis of mouse miRNAs (Agilent mouse miRNA microarray, based on MirBase release 12.0, and Affymetrix GeneChip miRNA array 1.0, based on MirBase release 11) were used to analyze and compare the miRNA expression pattern in RNA extracts derived from two different conditions, i.e. lung of wild type mice and lung of SP-C/c-raf mice expressing the c-Raf-1 transgene. Each gender and condition was tested separately using 6 different individuals from each group: male and female wild type mice (M WT and F WT) and male and female transgenic mice (M c-Raf and F c-Raf). MiRNA features which are commonly present on the two microarray platforms (“overlapping genes”) are 586.

The Affymetrix miRNA microarray platform detected the presence of 111 miRNAs in samples from WT male, 136 miRNAs in WT female, 141 in c-Raf male and 136 in c-Raf female. The Agilent miRNA microarray platform revealed the presence of 193 miRNAs in WT male, 239 miRNAs in WT female, 267 miRNAs in c-Raf male and 234 miRNA in c-Raf female.

**Figure 1:**
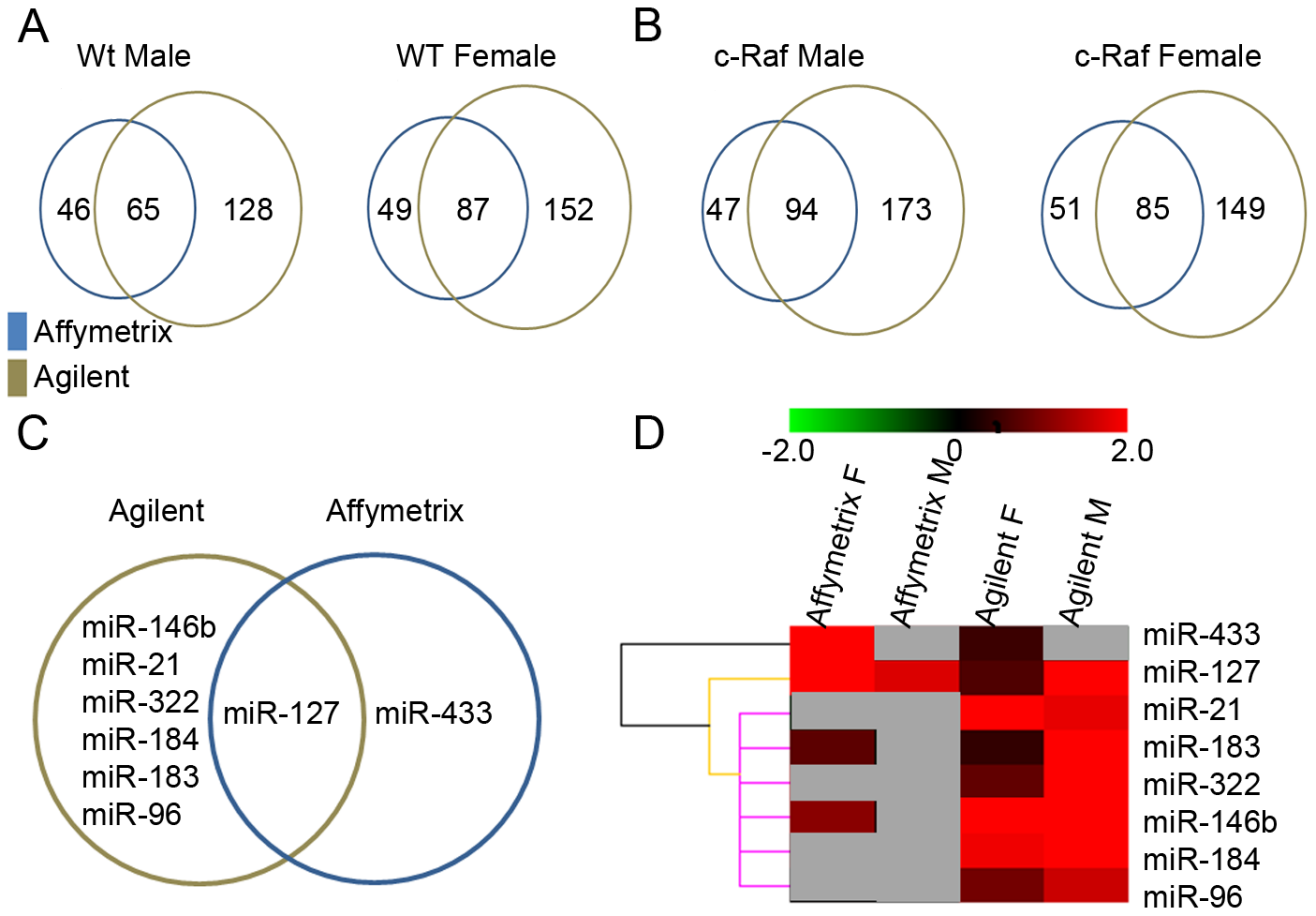
Discordant miRNA expression in lung cancer as determined by the Agilent and Affymetrix microarray platforms. **A)** Shown is the Venn diagram of detected miRNAs by the two different microarray platforms in male and female wild-type mice, respectively. **B)** Shown is the Venn diagram of detected miRNAs by the different microarray platforms in male and female c-Raf transgenic mice, respectively. **C)** Venn diagram for significantly regulated miRNAs as determined by the different microarray platforms. **D)** Hierarchical gene cluster analysis. The log2 ratio normalized data were used to perform the HCA analysis for differently expressed miRNAs in the lungs of male and female wild-type and c-Raf-transgenic mice. miR-433 was clearly segregated and found to be exclusively up-regulated in female transgenic mice as detected by the Affymetrix microarray platform. The dendogram is suggestive for miR127 to be regulated in common when both platforms are compared while a third group of miRNAs is regulated in common amongst male transgenic mice as detected by the Agilent platform.

The Venn diagrams in Figure 1 A and B summarize the number of miRNAs detected in the cross-platform comparison. The Affymetrix platform detected between 19% and 24% of the overlapping genes, while the Agilent platform detected 32–46% of the overlapping genes, depending on the sample. Between 11% and 16% of overlapping genes were commonly detected by the two platforms. When comparing samples from male and female mice, both platforms detected some gender-specific miRNAs.

To identify miRNAs significantly regulated as a consequence of c-Raf hyperactivity, the pairwise Welch’s t-test between transgenic and non-transgenic animal groups was applied. Table 1 summarizes miRNAs significantly regulated (≥1.5 Log_2_Ratio). With the Agilent platform significant up-regulation of, miR-21, miR-184 and miR-146b (borderline significant in male, Table 1) in male and female transgenic animals was observed, although at different levels in the two sexes. Moreover, miR-96, miR-183, miR-322 were significantly up-regulated in male c-Raf mice. Here miR-127 was highly up-regulated in male, but did not reach statistical significance (borderline at p<0.06; Table 1). The Affymetrix platform detected only the up-regulation of miR-127 in both female and male c-Raf transgenic mice (log_2_ Ratio = 2.57 for females and 1.73 for males), and of miR-433 only in female c-Raf (log_2_ Ratio = 2.10). Hence, much to our surprise, the two platforms correlated at best in identifying miR-127 as up-regulated in male, but not female, transgenic animals (Figure 1C).

**Table 1:** Significantly regulated miRNAs in male and female c-Raf transgenic mice.

	Affymetrix				Agilent				qRT-PCR			
miR	P value F	Log2	P value M	Log2	P value F	Log2	P value M	Log2	P value F	Log2	P value M	Log2
		(F_cRaf/F_WT)		(M_cRaf/M_WT)		(F_cRaf/F_WT)		(M_cRaf/M_WT)		(F_cRaf/F_WT)		(M_cRaf/M_WT)
mmu-miR-127	0.0003	**2.57**	0.0052	**1.73**	0.5827	**0.63**	0.0648	**4.18**	0.0005	**3.36**	0.0001	**2.44**
mmu-miR-146b	0.0306	**1.09**	0.3108	**0.2**	0.0138	**2.39**	0.0508	**3.79**	0.0067	**1.40**	0.0001	**0.58**
mmu-miR-183	0.3366	**0.74**	0.6165	−**0.12**	0.5505	**0.41**	0.0437	**2.15**				
mmu-miR-184	0.3369	**0.45**	0.1274	**0.40**	0.0397	**1.89**	0.0158	**4.09**	0.0001	**3.55**	0.0001	**2.68**
mmu-miR-21	0.6102	−**0.20**	0.7300	−**0.05**	0.0229	**2.06**	0.0369	**1.82**	0.0050	**1.95**	0.9380	**0.72**
mmu-miR-322	0.1733	**1.07**	0.3928	**0.25**	0.1520	**0.78**	0.0240	**2.82**	0.0004	**2.37**	0.0009	**1.21**
mmu-miR-433	0.0041	**2.10**	0.2029	**0.43**	0.7133	**0.47**	0.9387	−**0.13**	0.0001	**3.05**	0.0003	**1.96**
mmu-miR-96	0.7082	**0.09**	0.9150	**0.02**	0.0472	**0.91**	0.0180	**1.60**	0.0001	**2.37**	0.2514	**0.26**
mmu-miR-146a	0.0743	−**0.70**	0.3121	**0.38**	0.4024	−**0.13**	0.0357	**1.85**	0.0003	**1.40**	0.1445	**0.60**
mmu-miR-182	0.6368	**0.11**	0.3054	**0.14**	0.0433	**0.47**	0.1198	−**1.11**	0.0001	**2.19**	0.0059	**1.65**
mmu-miR-15a	0.8592	**0.08**	0.8222	−**0.06**	0.2448	**0.47**	0.0345	**1.43**	0.0001	**1.57**	0.2966	**0.40**
mmu-miR-34a	0.1684	**0.45**	0.3754	**0.51**	0.2185	**0.57**	0.0449	**1.44**	0.0001	**1.47**	0.0013	**1.02**

F = female, M = male, c-Raf = c-Raf transgenic lungs, WT = wild type.

The results are also visualized by the hierarchical clustering tree analysis (Figure 1D) performed with the Pearson Correlation. The majority of differentially expressed miRs identified by the Agilent platform were not detected as expressed by the Affymetrix microarry and the algorithm clearly segregates the data according to the two platforms used.

### Validation of Differentially Expressed miRNAs by Quantitative Real Time-PCR

Differential miRNA expression was examined by quantitative real time PCR (qRT-PCR) of the eight regulated miRNAs (miR-21, miR-96, miR-127, miR-146b, miR-183, miR-184 and miR-322, miR-433). We also analyzed miR-182, whose gene is in proximity to that of miR-96 and miR-183, and miR-146a which differs from miR-146b by only two bases (Table 2). In addition, we analyzed two miRNAs (miR-15a and miR-34a) whose levels had not been detected as significantly changed in c-Raf transgenic mice when compared to WT mice by both microarray platforms (Table 1).

**Table 2:** Sequences of miRNAs and probes used in microarray and qRT-PCR.

miRNA name	miRNA sequence	GCcontent(%)	Agilent			Affymetrix		
			probe sequences	free energy (kcal/mol)	Tm (°C)	probe sequence	free energy (kcal/mol)	Tm (°C)
**mmu-miR-15a**	UAGCAGCACAUAAUGGUUUGUG	40	CACAAACCATTATGTGCTGCT(A)	(−25.0)	56.2	CACAAACCATTATGTGCTGCTA	−25.0	56.2
				−24.4	56.9			
**mmu-miR-21**	UAGCUUAUCAGACUGAUGUUGA	36	TCAACATCAGTCTGATAAG(C)	(−20.3)	37.2	TCAACATCAGTCTGATAAGCTA	−22.7	39.9
				−17.6	32.0			
**mmu-miR-34a**	UGGCAGUGUCUUAGCUGGUUGU	50	ACAACCAGCTAAGACACTG(C)	(−23.2)	56.9	ACAACCAGCTAAGACACTGCCA	−27.7	60.4
				−20.5	52.8			
**mmu-miR-96**	UUUGGCACUAGCACAUUUUUGCU	40	AGCAAAAATGTGCTAGTGCCA(A)	(−24.1)	39.9	AGCAAAAATGTGCTAGTGCCAAA	−24.3	38.7
				−23.9	41.2			
**mmu-miR-127**	UCGGAUCCGUCUGAGCUUGGCU	59	AGCCAAGCTCAGACGGA(T)	(−23.2)	44.3	AGCCAAGCTCAGACGGATCCGA	−30.6	51.4
				−22.3	43.9			
**mmu-miR-146a**	UGAGAACUGAAUUCCAUGGGUU	40	AACCCATGGAATTCAGTT(C)	(−21.3)	55.6	AACCCATGGAATTCAGTTCTCA	−26.0	59.5
				−20	54.0			
**mmu-miR-146b**	UGAGAACUGAAUUCCAUAGGCU	40	AGCCTATGGAATTCAGTT(C)	(−21.5)	41.5	AGCCTATGGAATTCAGTTCTCA	−26.2	47.4
				−20.2	39.2			
**mmu-miR-182**	UUUGGCAAUGGUAGAACUCACACCG	48	CGGTGTGAGTTCTAC(C)	(−19.9)	62.9	CGGTGTGAGTTCTACCATTGCCAAA	−31.9	62.9
				−17	58.8			
**mmu-miR-183**	UAUGGCACUGGUAGAAUUCACU	40	AGTGAATTCTACCAGTGC(C)	(−23.2)	44.7	AGTGAATTCTACCAGTGCCATA	−26.3	46.3
				−20.3	40.0			
**mmu-miR-184**	UGGACGGAGAACUGAUAAGGGU	50	ACCCTTATCAGTTCTCCGTCC(A)	(−31.9)	57.0	ACCCTTATCAGTTCTCCGTCCA	−31.9	57.0
				−30.3	56.2			
**mmu-miR-322**	CAGCAGCAAUUCAUGUUUUGGA	40	TCCAAAACATGAATTGCTGCTG	−23.1	37.7	TCCAAAACATGAATTGCTGCTG	−23.1	37.7
**mmu-miR-433**	AUCAUGAUGGGCUCCUCGGUGU	54	ACACCGAGGAGCC(C)	(−20.7)	43.7	ACACCGAGGAGCCCATCATGAT	−30.3	50.9
				−17.8	38.0			

Free energy for the probe-target binding was calculated using the thermodynamic parameters for RNA/DNA hybrid duplexes from Sugimoto et al. [69]. Tm was calculated according to the formula reported in Wei et al. [70] using the thermodynamic parameters for RNA/DNA hybrid duplexes from Sugimoto et al. [69] that consisted of the following parameters: [Na+] = 1 M, Ct (oligonucleotide concentration) = 100 uM, A (helix initiation factor) = −10.8 cal/KM, F (correction term per1% Formamide) = 0.63°C, Formamide concentration = 35%, R (universal gas constant) = 1.987 cal/KM.

**Figure 2:**
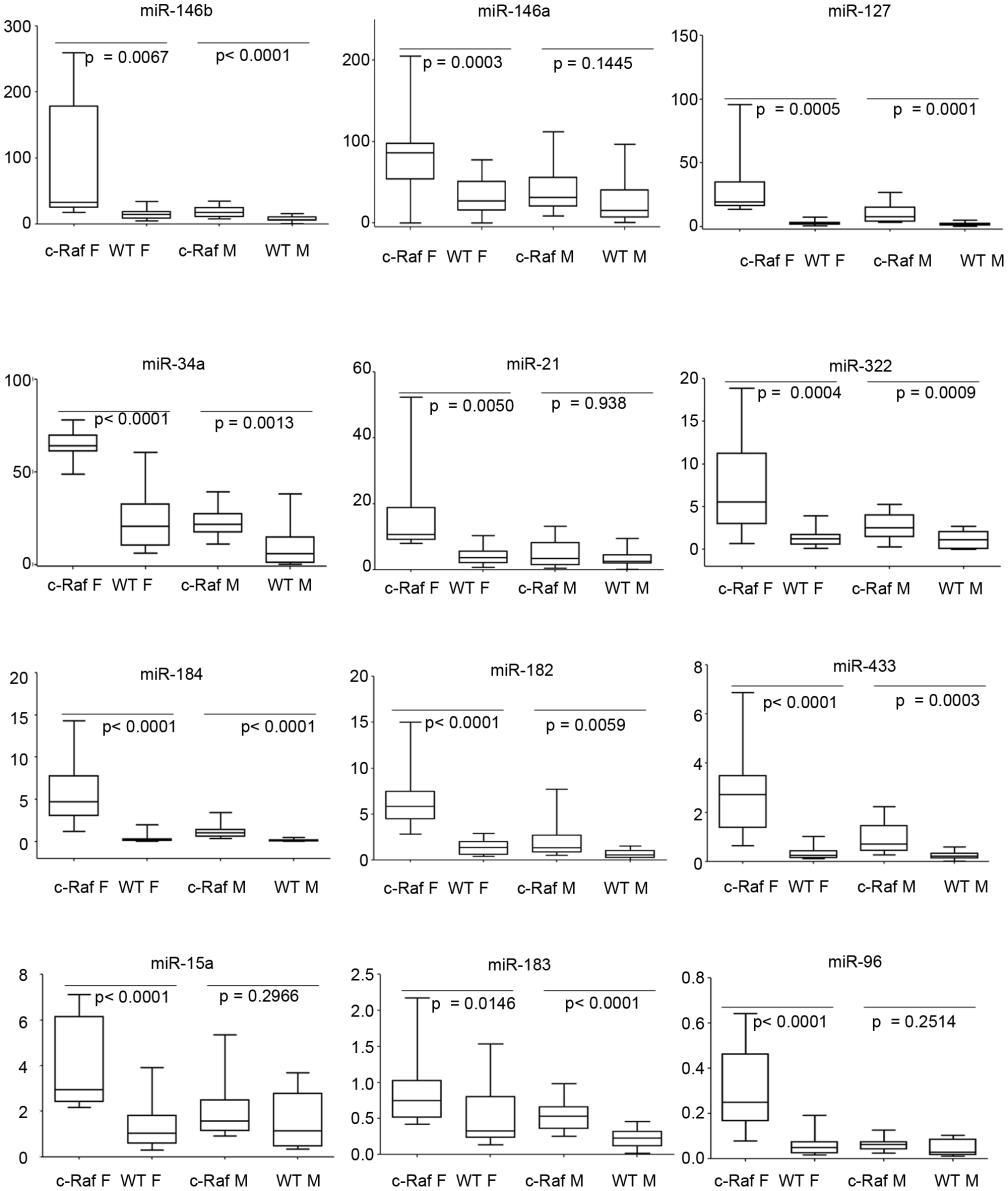
Quantitative real time PCR of differentially expressed miRNAs. Shown is the expression of miR-21, miR-146b, miR-127, miR-433, miR-96, miR-183, miR-184 and miR-322 in WT and transgenic male and female mice. Note, miR-182 is expressed as a cluster with miR-96 and miR-183 and share similar sequences. Therefore, this miRNA was included in the analysis even though it was unchanged in transgenic mice. Similarly, miR-146a differs by only one base from miR-146b and therefore was analyzed as well while miR-15a and miR-34a were included in the assay for not being regulated in c-Raf transgenic mice and thus served as controls.

The levels of miRNAs were measured and normalized to small nuclear RNA U6, every condition was measured for 6 biological replicates and every measurement was performed in triplicate. The results of the qRT-PCR for the 12 miRNAs are summarized in Table 1 and Figure 2. The inter-individual variation in the miRNA expression levels is considerable with high standard deviations particularly noted for c-Raf females (Figure 2). The qPCR results confirmed the significant over-expression of miR-127 and miR-433 in male and female c-Raf mice as identified by the Affymetrix microarray. However, a significant increase of miR-433 in male c-Raf was also observed that had not been revealed in the array platform. Only the Agilent data for miR-184 and miR-146b, as well as for miR-21 (here female mice), were confirmed by the qPCR. The gender bias for miR-183 and miR-322 was not confirmed, but the two miRs were up-regulated in all transgenic animals. An opposite gender bias was found for miR-96 in male c-Raf. Finally, qPCR detected a significant increase of miR15a and 146a in females and of miR-182 and miR-34a in both genders, which had not been observed by the microarray platforms.

### MiRNA Target Prediction and Functional Characterization of Predicted miRNA Targets

**Figure 3:**
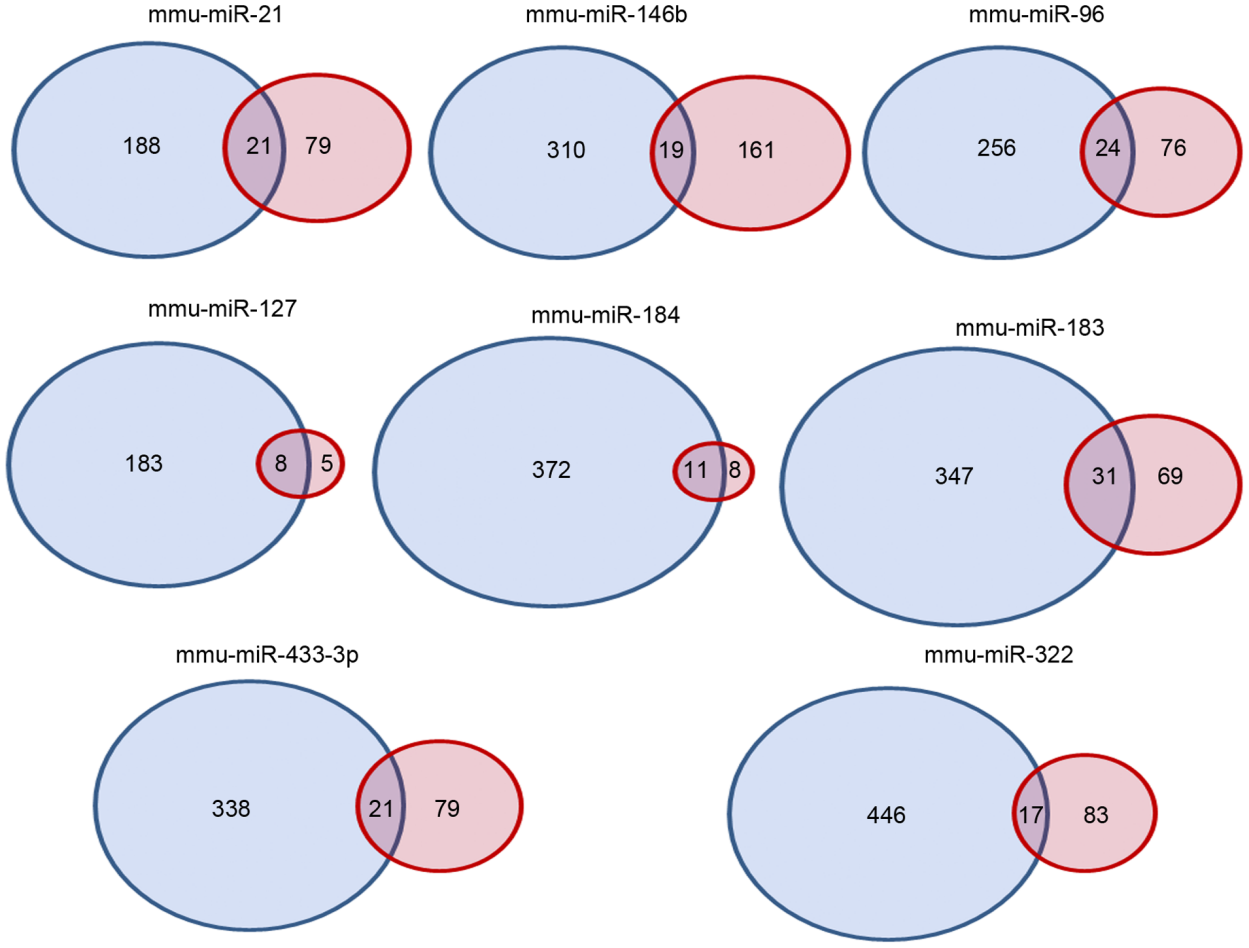
Target gene prediction based on the software PITA (blue circles) and Targetscan (red circles). Depicted is a Venn diagram of predicted target genes using the two different software.

To further explore the biological significance of regulated miRNAs information from both microarray platforms was combined with the aim to functionally characterizate them. We predicted the target mRNAs by using the webtools Targetscan (http://www.targetscan.org/) and PITA (http://genie.weizmann.ac.il/pubs/mir07/index.html). Both miRNA target prediction programs rely on sequence complementarities of the miRNA seed region (nucleotides 2–7) to the 3′UTR sequences in candidate target genes. However, TargetScan [35] makes use of evolutionary conservation at the binding site in order to minimize false positives, and takes into account proximity of one binding site to another within the same 3′ UTR [36]. The PITA Algoritm, on the other hand, is based on the structural accessibility of the binding site, computing the secondary structure of the miRNA::target mRNA hybrid molecule and the overall thermodynamics of the interaction expressed in free energy (dG) [37]. The results of the two analyses are summarized in the Venn diagram depicted in Figure 3 (see also Table S1 for detailed information on predicted target genes).

The list of the predicted targets was compared with that of the 64 genes significantly regulated in transgenic but unaltered lung tissue as was previously reported [28]. Here, no match was obtained. Then, the list of putative target genes was compared with n = 249 genes significantly regulated in laser-dissected lung dysplasia of c-Raf-transgenic mice we likewise reported in 2009 [27]. Here, 4 matches were found (AZIN1, CHST1, CRISPLD2 and FOXQ1). Lastly, we compared putative target genes with the set of significantly regulated genes in laser-dissected lung adenocarcinomas of c-Raf-transgenics [n = 370 genes, ref. 28] obtaining a list of 11 genes, i.e. CHST1, CRISPLD2, GATA3, GPC3 HPGD, PCDh1, PRICKLE1, RECK, RUNTX1T1 and SPOCK2. All of these 11 were repressed at the mRNA level with fold changes ranging from 0.04 to 0.17-fold (Table 3).

**Table 3:** Transcriptional regulation of genes targeted by regulated miRNAs in c-Raf transgenic mice.

Gene Name	Displasia FoldChange	Adenocarcinoma FoldChange	Percentage (%) of tumorsamples show more signalthan pneumocytes	Percentage (%) of tumor samples show less signal than pneumocytes
azin1	4.82	–	0	92
foxq1	6.98	–	N/A	N/A
pcdh1	–	0.17	N/A	N/A
runx1t1	–	0.17	N/A	N/A
reck	–	0.15	0	100
crispld2	0.2	0.11	0	92
gpc3	–	0.11	21	47
spock2	–	0.10	55	0
gata3	–	0.08	0	50
chst1	0.15	0.07	N/A	N/A
prickle1	–	0.07	0	0
hpgd	–	0.04	16	67

Given is the repression of target genes at the mRNA level in lung dysplasia [27] and lung adenocarcinoma [28]. The first column indicates the fold changes of genes repressed in lung dysplasia in c-Raf transgenic mice [27]. The second column indicates the fold changes of mRNAs repressed in lung adenocarcinomas from c-Raf mice. For each gene the percentage of immunohistochemistry signal intensity for lung tumors as compared to healthy surrounding tissue is given and is based on data reported in the Human Protein Atlas database. N/A = Non-Available data.

To determine the clinical significance of our findings the protein expression of these genes in human lung cancer was analyzed using data of the publically available protein atlas repository (www.proteinatlas.org). In the case of RECK, CRISPLD2, HPGD, GATA3 and GPC3, the signal related to the expression of the protein was down regulated in more than 45% of adenocarcinomas compared to surrounding healthy pneumocytes (see Table 3). This demonstrates the relevance of the transgenic disease model for human lung malignancies.

### Functional Characterization of Predicted miRNA Gene Targets in Human Lung Cancer

**Figure 4:**
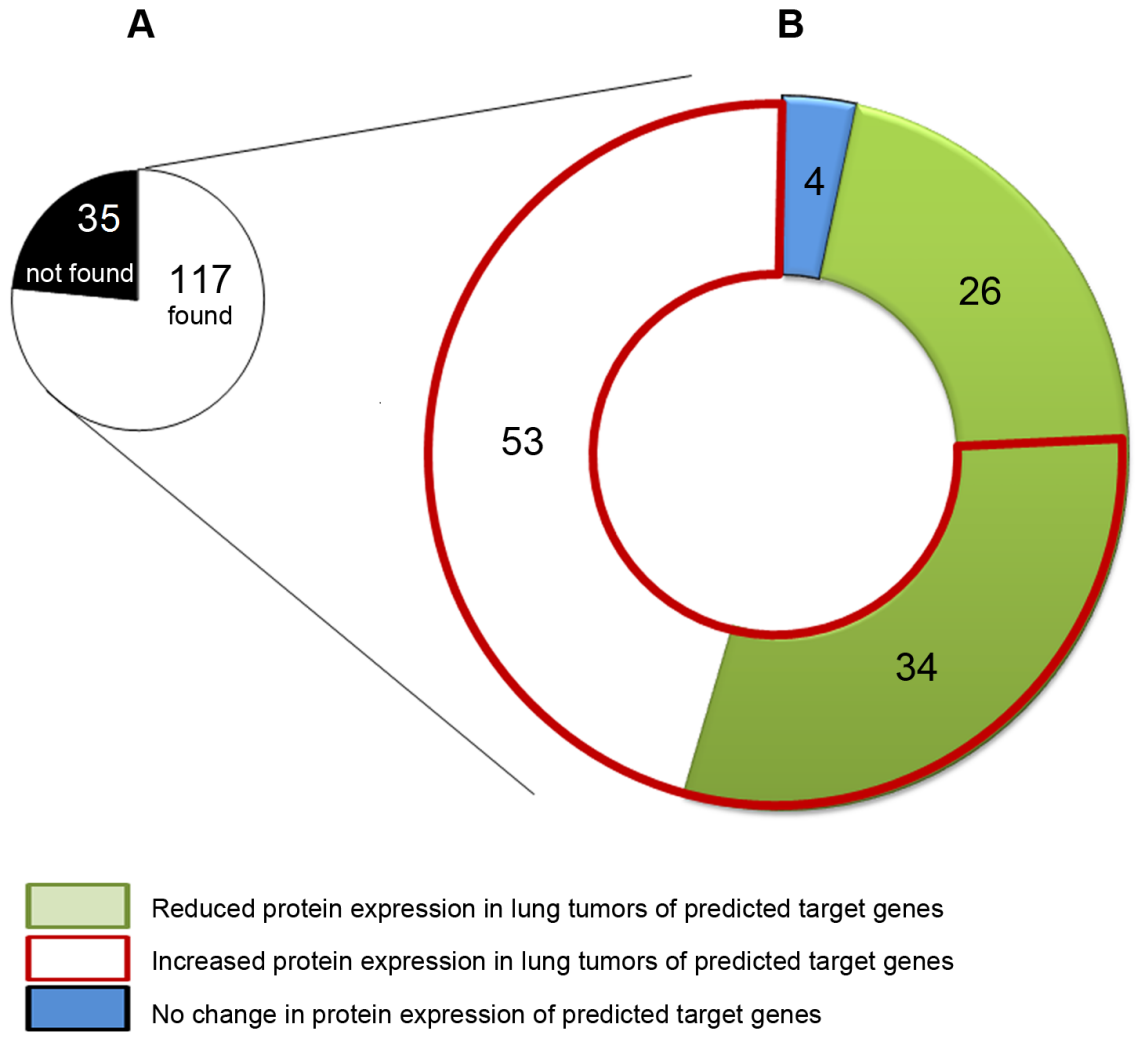
Functional characterization of predicted miRNA target genes in human lung cancer. For 8 significantly regulated miRNAs 152 human target genes were predicted by the TargetScan and PITA software. Subsequently, their protein expression in lung tumors was assessed using the publically available Human Protein Atlas database. **A.** The pie chart informs on the number of genes with information on their expression as determined by immunohistochemistry in healthy human lung and lung cancer. **B.** For 117 genes expression of the coded protein in healthy lung and lung tumors is given, and the data for immunohistochemisty (IHC) staining was analyzed by comparing signals from tumors and surrounding normal tissue. A list of all target genes predicted for individual regulated miRNA is given in Table S1. Specifically, for each gene the signal as determined by IHC was calculated for healthy and tumor tissue and grouped into various categories as depicted. The individual data are given in Table S1.

To further validate the findings, expression of putative target genes was assessed at the protein level using the data of the Human Protein Atlas repository, version 9.0 [http://www.proteinatlas.org/]. The pie chart depicted in Figure 4 provides information on the number of putative gene products found in the data base and their expression in human lung malignancies. While no information was available for 36 gene products, data could be retrieved for 117 predicted target genes using the algorithm described above. For 26 gene products a lower, for 53 genes an increased staining and for 34 genes variable results were determined while for 4 genes no change in expression of proteins in human lung cancer was noted. Overall, the data agree well with the presumed activity of the regulated miRNAs found in lung tissue of c-Raf transgenic mice; the complete information on the data analysis is given in Table S1.

**Figure 5:**
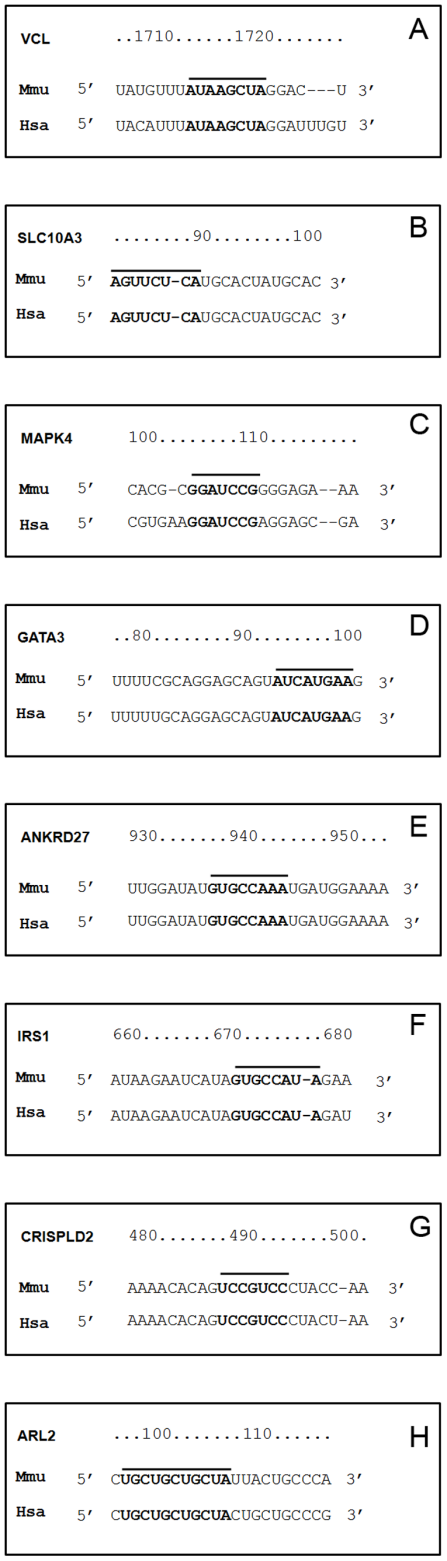
3′ UTR seeding sites of regulated miRNAs in mouse and human orthologous genes. The 3′UTR sequence alignment of VLC, SLC10A3, MAPK4, GATA3, ANKRD27, IRS1, CRISPLD2 and ARL2 between *Mus musculus* and *Homo sapiens* species may possibly suggest conservation of seed sequences targeted by miR-21 (panel A), miR-146b (panel B), miR-127 (panel C), miR-433 (panel D), miR-96 (panel E), miR-183 (panel F), miR-184 (panel G) and miR-322 (panel H), respectively.

Finally, Figure 5 informs on the commonality in the 3′UTR seeding sequence of miRNAs in orthologous mouse and human genes to possibly suggest evolutionary conservation.

**Figure 6:**
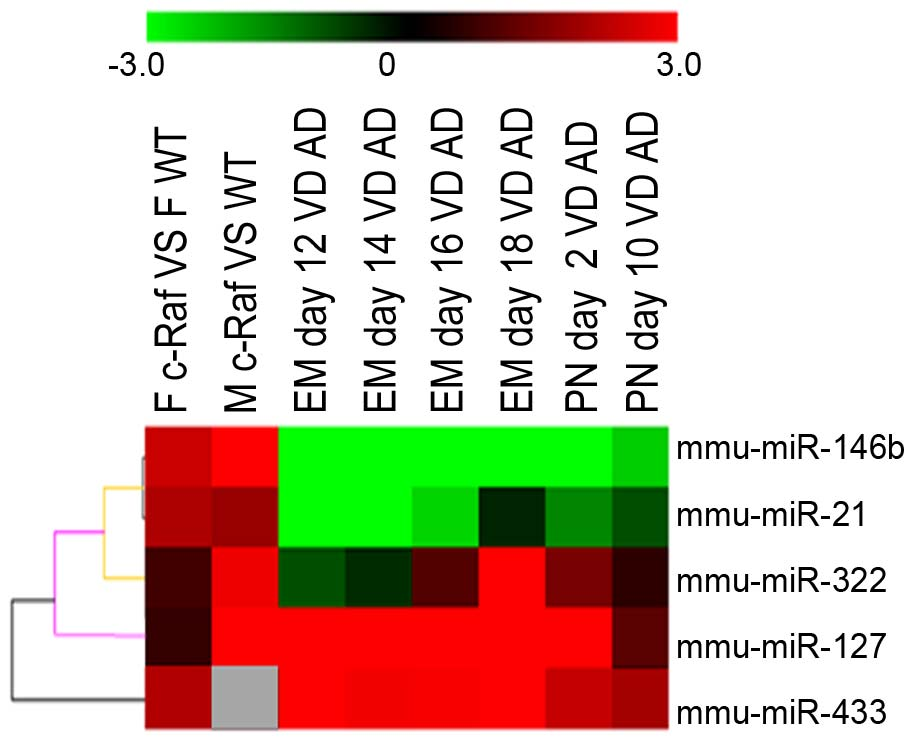
miRNA expression in lung development and lung cancer. Hierarchical gene cluster analysis evidences co-regulation of miR-21 and miR-146b during organogenesis and in lung cancer. Both oncomiRNA were up-regulated in c-Raf transgenic lung but were repressed during lung organogenesis [56], [57]. miR-322, miR-127, and miR-433 are regulated similarly in organ and tumor development.

Data of the present study was also compared with recently published miRNA profiles for mouse lung development [38]. In this study 117 miRNAs were shown to be differentially regulated throughout organogenesis (6 time points starting from E12 to day 2 post partum). Of these five were also significantly regulated in c-Raf transgenic lungs as summarized in Table 4. Moreover, hierarchical clustering depicted in Figure 6 points to a co-regulation of miR-21 and miR-146b. Both oncomiRNA were up-regulated in c-Raf transgenic lung but were repressed during lung organogenesis. The remaining 3 miRNAs were regulated similarly in organ and tumor development.

**Table 4:** Expression of selected miRNA in lung development and lung cancer.

miRNA	F C-Raf VSF WT	M C-Raf VSM WT	Em Day12 VS AD	Em Day14 VS AD	Em Day16 VS AD	Em Day18 VS AD	PN Day2 VS AD	PNDay10 VS AD
mmu-miR-127	0.63	4.18	4.06	3.44	4.47	5.53	3.41	1.06
mmu-miR-146b	2.39	3.79	−6.91	−7.51	−6.49	−3.92	−3.57	−2.42
mmu-miR-21	2.06	1.82	−3.95	−3.04	−2.51	−0.44	−1.58	−0.93
mmu-miR-322	0.78	2.82	−0.94	−0.52	0.98	3.03	1.42	0.56
mmu-miR-337-5p	2.08	7.22	3.49	3.39	3.42	4.49	2.86	0.61
mmu-miR-433	2.10	0.43	3.48	2.89	2.91	4.47	2.35	1.97

miRNA expression data for lung organogenesis stems from [26]. F = female, M = male, c-Raf = c-Raf transgenic lungs, WT = wild type, Em Day = Embrionic Day, PNDay = Post Natal Day, AD = Adult. Data are given as fold change.

**Figure 7:**
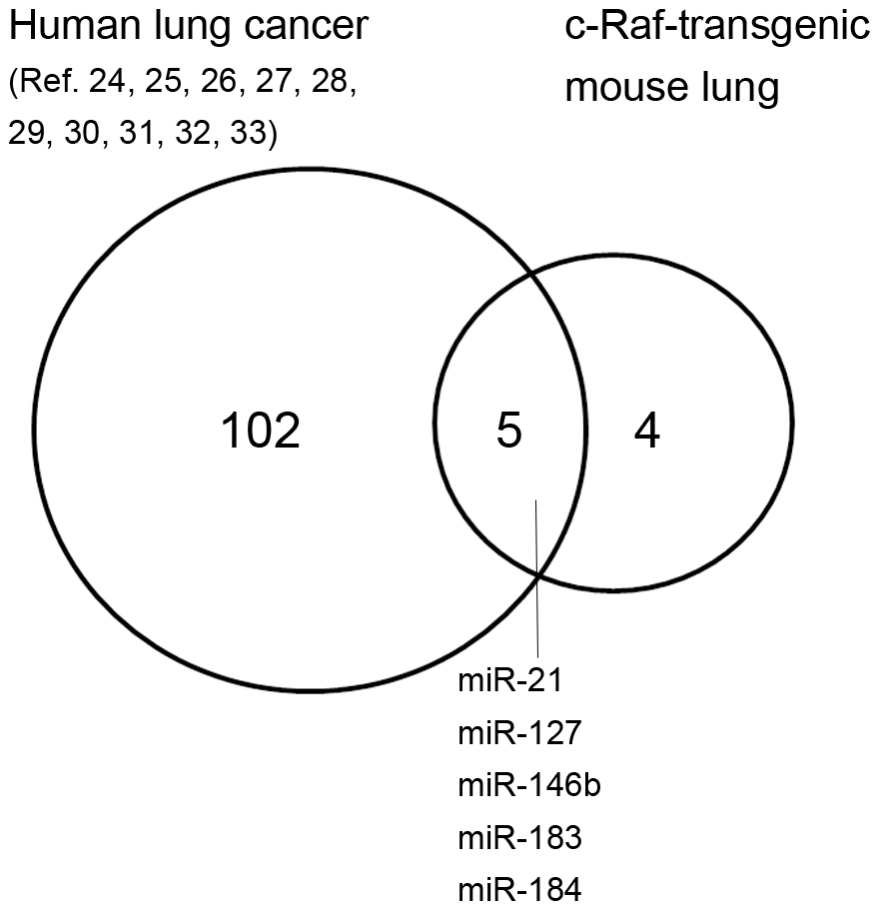
miRNA expression profiling in human lung cancer and c-Raf transgenic mice. Eight significantly regulated miRNAs identified in c-Raf transgenic mice were compared with the list of 107 miRNAs reported as regulated in human lung cancer studies [39], [48]. Five miRNAs, miR-127, miR-21, miR-146b, miR-183, miR-184 were similarly up-regulated in c-Raf transgenic lung and human lung cancer therefore demonstrating clinical relevance of this particular disease model.

We compared the list of 8 miRNAs differently expressed in the lung of c-Raf-transgenic mice, with the list of 107 miRNAs compiled from the results of studies in human lung cancer [39]–[48]. Five miRNAs, miR-127, miR-21, miR-146b, miR-183, miR-184 were similarly up-regulated in c-Raf transgenic mouse lung and human lung cancer thus further validating this model as relevant for human lung cancer (Figure 7).

## Discussion

For their important role in the control of gene expression, in mRNA stability as well as ribosomal translation, small RNAs are the subject of intensive research. So far more than 1000 miRNAs were identified in mouse and human tissues with single miRNAs targeting multiple genes. Complete information on the regulation of all known miRNAs is therefore needed which can be obtained by the use of enabling technologies. While several technologies are available to enable genome-wide scans, assay differences amongst platforms need to be rigorously characterized. Importantly, in the recent study by Git and colleagues [14], six different microarray platforms in addition to new generation sequencing and qRT-PCR were compared, evidencing considerable discrepancies among the miRNA expression levels for the same sample. Thus, caution must be taken when data obtained from different platforms are compared, taking sample preparation and technology dependent data collection into account. However, a cross-validation of miRNA profiling with the Agilent and Affymetrix platform has not been reported so far. In the first part of our study we searched for disease regulated miRNAs in a c-Raf transgenic mouse model of lung cancer. This model was extensively examined in our laboratory and by other investigators. In the past we reported regulation of genes in precursor lesions and malignantly transformed epithelium that had been the subject of independent reports (see [15], [16]). Based on a wealth of gene expression data we initially investigate the technical reproducibility in miRNA profiling among the Agilent and Affymetrix platforms and employed quantitative real-time PCR as an independent method to validate individual results as summarized in Table 1.

While for some of the miRNAs the data compared reasonably well across the platforms, the p-values for some regulated miRNAs differed considerably. Specifically, with the Agilent platform a significant regulation of miR-21, miR-96, miR-127, miR-146b, miR-183, miR-184 and miR-322 was observed whereas for the Affymetrix platform significant regulation of miR-127 and miR-433 could only be evidenced. Moreover, the expression of miRNAs was in the order of 3 to 8 log_2_Ratio with a total of 6 and 2 miRNAs being statistically significantly regulated amongst the two microarray platforms compared. The significant differences in the detection of regulated miRNAs may possibly be linked to the differences of the sequences spotted onto the microarray. Specifically, miR-433 was not identified as differentially expressed when analyzed with the Agilent platform while its expression was determined as statistically significantly up-regulated with the Affymetrix microarray and by qRT-PCR. In contrast, the miR-21 and miR-146b were found as significantly regulated on the Agilent microarray and by qRT-PCR but not with the Affymetrix platform.

The two microarray platforms used differ in regards to several respects. Specifically, with the Agilent platform microRNAs are dephosphorylated, a C-residue is added at the 3′ end and labeling is performed by ligation of Cy3; however, with the Affymetrix platform the microRNAs are labeled by polyadenylation and ligation of the 3′ to a biotinilated DNA.

Moreover, the hybridization conditions differ amongst the two platforms, i.e. the hybridization mix and the conditions for arrays differ that may adversely affect data generation.

Finally, with the Affymetrix chips, oligonucleotide probes up to 25-mers are spotted, with perfect complementarities to the mature miRNAs. However, the Agilent microarrays employ 40–60 mers with a 5′terminal hairpin to destabilize interactions with the pre-miRNA, and a 3′ portion of 10–30 nucleotides complementary to the mature microRNA.

We reasoned that sequence differences of the employed probes could be a possible cause for the platform-dependent findings. Thus, Table 2 informs on the sequences employed for the miRNA detection. While it is evident that the Agilent probe for miR-433 is shorter than the other probes, the calculated deltaG and Tm are not dissimilar from the ones of the miR-21 probe. Therefore it is not easy to explain, based on chemico-physical parameters, why miR-433 variation is not detected by the Agilent platform. Similarly, it is not clear why Affymetrix fails to see variations in all but two miRs (-127 and -433), since for example the probe for miR-184 has similar parameters to those for miR-127 and miR-433.

In a study comparing Affymetrix and Agilent platforms Sah and co-authors [20] reported that both demonstrated good accuracy but with less precision than Illumina and Exiqon microarray platforms. However, Pradervand and colleagues [21] have shown divergence among the Affymetrix, Agilent and Illumina microarray platforms, and good overall concordance was achieved among the Agilent microarray, TaqMan qRT-PCR and Illumina ultra high-throughput sequencing technology. The authors concluded that the Agilent platform outperforms the Illumina and Affymetrix platforms due to its greater accuracy in fold change measurement and its accurate profiling of miRNAs that differ in GC content. In fact, they observed that in the Affymetrix platform the mean GC content of false negatives was significantly lower than the GC content of the true positives (42.4% vs 50.5%). It is interesting to note that in the present study the GC contents of the two miRNAs detected as deregulated by Affymetrix are higher (54% and 59%) than the GC content of the miRNAs that Affymetrix failed to detect as deregulated (36–50%; see Table 2).

In the study of Leshkowitz and colleagues [25] the Affymetrix miRNA 2.0 and Agilent microarray platforms were compared. Again, the detected miRNA expression levels were not consistent between platforms, and the authors concluded that miRNA base composition, sequence structure and isoforms impact on the detection levels. Notably, using spike-in samples the authors could demonstrate with the Affymetrix platform the erroneous detection of abundant precursor miRNAs as mature miRNAs that was not seen with the Agilent platform. Moreover, they reported that the Affymetrix array suffers from an over-representation of guanine-rich miRNAs and from an under-representation of uracil-rich miRNAs and had a lower sensitivity for those miRNAs that have more isoforms, mismatches, and 5′insertions. The Agilent array was more permissive to mismatches and 5′ insertions, possibly due to the probe design which connects the probe to the glass surface by a linker and includes a hairpin and an additional guanine, both possibly stabilizing miRNA binding.

In another recent paper, Kolbert and co-authors [26] reported that detection of miRNAs from lung tissues and from lung cancer cell lines was similar across Affymetrix, Agilent and Illumina microarray platforms, and that Affymetrix exhibited the highest correlation with the qRT-PCR assay used (Fluidigm BioMark System). Relevant to our results, these authors reported that in a pairwise comparison between Agilent Human v2 array and Affymetrix 1.0 array, Affymetrix detected 35–45% of overlapping genes, Agilent detected 36–38% of overlapping genes and about 35% of overlapping genes were commonly detected by both platforms when fresh-frozen human lung tissues were analyzed (see Supplementary Tables 1 and 2, provided in [26]).

Similarly to Predervand [21] and Leshkowitz [25], we suggest that the discordance we observe between Agilent and Affymetrix might be due to the probe design, rather than the labeling procedures, which are similar. Moreover, our results are in line with previous reports [18], [19], [21], [22] which show good concordance between Agilent and qRT-PCR data.

Importantly, miR-21 over-expression is largely associated with tumor development [49] while miR-146b over-expression was reported for the human lung cancer cell line A549 [50] and in T-cells from patients with severe asthma [51]. Moreover, miR-146a and miR-146b have been shown to play a central role in the negative feedback regulation of IL-1b-induced inflammation; the mechanism is down-regulation of two proteins IRAK1 and TRAF6 involved in Toll/interleukin-1 receptor (TIR) signalling [52], [53]. In our previously published cancer genomics study TRAF2, another member of the TNF receptor associated factors, was highly significantly repressed by more then 80% but IRAK1 was unchanged in c-Raf transgenic lung cancers [28].

Furthermore, miR-21 is crucially involved in allergic lung inflammation. Its molecular target is IL-12p35, a cytokine contributing to polarization of Th cells toward Th2 cells [54]. miR-21 was also reported to be up-regulated in squamous cell carcinoma [55] while miR-127, miR-322 and miR-146b are up-regulated during fetal lung development [56], [57]. Importantly, miR-183 and miR-96 belong to the same locus, located on chromosome 6 and 7 in mouse and human, respectively. Furthermore, miR-433 and miR-127 are within the same locus, and they are co-transcribed and co-regulated by estrogen-related receptors gamma [58]. Evidence has also been obtained to suggest a conserved gene structure and transcriptional regulation of miR-433 and miR-127 in mammals and that the miR-433/127 loci may have evolved from a common gene of origin. Lastly, miR-127 was hypothesized to be involved in alveolar septation by inhibiting mRNA translation of specific targets [38].

Next to the cross-platform comparison we investigated a possible link between c-Raf hyperactivity and regulation of miRNAs in c-Raf transgenic mice displaying precursor lesions, i.e. atypical adenomatous hyperplasia of the lung. As described above miR-21 was significantly up-regulated in qRT-PCR experiments and when studied with the Agilent microarray. This miRNA can be activated by STAT3 [59] via PTEN and cyclin D and is part of an epigenetic switch linking inflammation to cancer [60]. Moreover, STAT 3 dependent serine phosphorylation by insulin has been reported to be mediated via a Ras/Raf/MEK-dependent pathway [61]. Finally, activation of different Ras effectors was necessary for miR-21 induction in fibroblasts [62]. It is tempting to speculate that up-regulation of miR-21 is STAT3 dependent even though our previously published proteomic analyses [32], [33] did not reveal induction of STAT transcription factors in c-Raf transgenic mice. However, post -translational modifications and phosphorylation cascades were not specifically investigated in this study.

For 152 gene products predicted to be target of the 8 miRNA indentified in the present study, a gene ontology analysis was carried using the Babelomics web-tool version 4.3 [http://babelomics.bioinfo.cipf.es/functional.html]. The molecular function analysis showed enrichments in cation transmembrane transporter activity (10.53%, 3.5-fold, *p* = 0.0036) and ion transmembrane transporter activity (11.84%, 3.1-fold, *p* = 0.0036). Note, there is growing evidence for some cation transporter not only to be an independent predictor of chemotherapy response but also to function as a prognostic factor in stage III NSCLC. In contrast, cellular component analysis did not reveal enrichment with satisfying *p*-value while classification of biological processes evidenced regulation in metal ion transport (11.84%, 4.8-fold, *p*<0.001).

We also employed various bioinformatic tools for an identification of possible target genes in precursor lesions and solid lung tumors of the c-Raf transgenic mice. We first compared the list of putative target genes predicted for the regulated miRNAs with the results obtained for c-Raf transgenic mice [28], [29]. Of the 152 predicted gene targets in mouse and human genomes, four were significantly regulated in laser dissected lung dysplasia (AZIN1, CHST1, CRISPLD2 and FOXQ1) while 11 genes were significantly regulated in laser-dissected adenocarcinomas (Table 3). Of the repressed target genes RECK is an important example. This membrane-anchored matrix metalloproteinase-regulator is frequently down regulated in cancers and a significant correlation exists between the level of residual RECK expression in resected tumors and patient survival [63].

To further corroborate findings, data from the Human Protein Atlas, version 9.0 [http://www.proteinatlas.org/] was compared with the list of predicted gene targets identified in the cancer genomics studies we previously published. The pie chart shown in Figure 4 provides information on the number of putative gene products found in the data base and their expression in human lung malignancies. While no information was available for 36 gene products data could be analyzed for 117 proteins of which 26 show strong repression in the lung tumor samples, whereas 34 were moderately repressed as determined by immunohistochemistry staining. These data agree well with the presumed activity of the regulated miRNAs in lung tissue of c-Raf transgenic mice.

For the 60 gene products predicted to be targets of the regulated miRNAs expression was repressed in lung tumors of c-Raf transgenic mice. Using the Babelomics web-tool version 4.3 [http://babelomics.bioinfo.cipf.es/functional.html] the gene ontology analysis was repeated and the molecular function analysis suggested enrichments in transcription co-factor activity (11.67%, 7.6-fold, p = 0.001) and protein kinase activity (3.33%, 37-fold, p<0.005). Cellular component analysis revealed enrichment for membrane fraction (18.33%, 5.14-fold p<0.001) while classification of biological processes evidenced regulation in cell transcription from RNA polymerase II promoter (13.33%, 3.65-fold, p<0.001), and negative regulation of metabolic process (13.33%, 4.50-fold, p<0.001).

We finally compared data for c-Raf transgenic mice with developmentally regulated miRNAs. In the recent study of Dong et al. [38] 117 miRNAs were shown to be significantly regulated throughout organogenesis. Of these 5 miRNAs were significantly regulated in c-Raf transgenic lungs (Table 4). The hierarchical clustering depicted in Figure 6 points to a co-regulation of miR-21 and miR-146b. Notably, while both oncomiRNA were up-regulated in c-Raf transgenic lung their expression was repressed during lung organogenesis. We also compared published data for regulated miRNAs in human lung cancer and observed five miRNAs to be similarly up-regulated in the comparison c-Raf transgenic mouse lung and human lung cancer to further demonstrate relevance of this transgenic model for human lung cancer (Figure 7).

In conclusion, the present study evidences significant differences in a cross-platform comparison that will have important implications for research into miRNAs.

## Materials and Methods

### SP-C/c-raf Model

SP-C/c-raf transgenic mice were obtained from the laboratory of Prof. Ulf Rapp (University of Würzburg, Germany). Lung tissue from n = 6 male and n = 6 females SP-C/c-Raf transgenic mice (aged 4–6 months) and n = 6 male and n = 6 female normal lung tissue from type mice (aged 4–6 months) were sacrificed and the lung tissues were immediately frozen on dry ice and stored at - 80° until further analysis. Prior to miRNA analysis lung tissue was also examined by histopathology using the hematoxylin-eosin stain.

All animal work followed strictly the Public Health Service (PHS) Policy on Human Care and Use of Laboratory Animals. Formal approval to carry out animal studies was granted by the Lower Saxony State Office for Consumer Protection and Food Safety (Niedersächsisches Landesamt für Verbraucherschutz und Lebensmittelsicherheit, LAVES, Hannover), Germany, reference number 33-42502-06/1081. The samples analyzed in the present study stem from recently conducted work as referenced in [27], [28].

### Microdissection (LMPC – Laser Microbeam Microdissection and Laser Pressure Catapulting)

From each frozen lung tissue 10-mm thick sections were prepared and transferred on polyethylene napthalate foil-covered slides (Zeiss, P.A.L.M. Microlaser Technologies GmbH, Bernried, Germany). The sections were fixed in methanol/acetic acid and stained in hematoxylin. The desired cells were microdissected using the PALM MicroLaser systems (Zeiss, P.A.L.M. Microlaser Technologies GmbH, Bernried, Germany) and collected in an adhesive cap (Zeiss, P.A.L.M. Microlaser Technologies GmbH, Bernried, Germany). Microdissected cells were resuspended in a guanidine isothiocyanate-containing buffer (RLT buffer from RNeasy MikroKit, Qiagen, Santa Clarita, CA, USA) with 10 µl/ml ß-mercaptoethanol to ensure isolation of intact RNA. Approximately an area of 66106 mm2 were pooled from a specific layer of interest in the same animal and used for RNA extraction. Following microdissection, total RNA-extraction was performed with the RNeasy Micro Kit (RNeasy MicroKit Qiagen, Santa Clarita, CA, USA) according to the manufacturer’s instruction. A standard quality control of the total RNA was performed using the Agilent 2100 Bioanalyzer (Agilent Technologies, Palo Alto, USA).

### RNA Labeling and Microarray Hybridization

#### Affymetrix microarray

From each lung 200 ng of total RNA was isolated and prepared for labeling of miRNA with the FlashTag Biotin HSR Procedure (Genisphere, Hatfield, PA, USA) according to the manufacturer’s instructions (http://media.affymetrix.com/support/downloads/manuals/mirna_flashtag_manual.pdf).

The samples were then hybridized to Affymetrix GeneChip miRNA array 1.0 that contained 722 and 690 mouse mature and pre-miRNA, respectively using a protocol recommended by the manufacturer (http://media.affymetrix.com/support/downloads/manuals/flashtag_user_guide.pdf).

The expression signals were processed and normalized by the quantile method using the miRNA QC Tool version 1.0.33.0 (Affymetrix), which was also used to check the quality of the labeling and hybridization (http://media.affymetrix.com/support/technical/datasheets/miRNA_2_datasheet.pdf).

#### Agilent microarray

One hundred nanograms of total RNA samples was dephosphorylated, 3′ end-labeled with Cy3-pCp, purified on Micro Bio-Spin columns, dried, and hybridized onto arrays using the miRNA Microarray System labeling kit V2 according to the manufacturer’s instructions (**5190-0456**) Arrays used for hybridization were Agilent mouse miRNA microarray (Release 12.0, catalogue ID G4472B) that contained 612 mouse mature miRNA features, each one in 20 technical replicates. Hybridized microarray slides were scanned with an Agilent DNA Microarray Scanner G2505C and data was analyzed with the Agilent ScanControl version 8.1.3 software. The scanned TIFF images were analyzed numerically and background corrected using the Agilent Feature Extraction Software version 10.7.7.1.

#### Data deposition

The Affymetrix and Agilent data are available at NCBI’s Gene Expression Omnibus (GEO) (http://www.ncbi.nlm.nih.gov/geo) under accession number GSE 50753.

#### Data Analysis

In the case of the Affymetrix miRNA arrays data analysis was done according to the instructions given by the manufacturer in the users guide, i.e. mirna-1_0_2xgain (http://media.affymetrix.com/support/downloads/manuals/mirna_qctool_user_manual.pdf).

The raw data obtained from the Agilent mouse miRNA microarrays were normalized to the 75th percentile signal intensity as recommended by the vendor. After normalization all negative signal values were replaced by 0.01 and the values from multiple replicate spots for each miRNA were summarized as median signals which were used subsequently for statistical analyses.

To identify significantly regulated miRNAs the pairwise Welch’s T-test between c-Raf-transgenic and non-transgenic animal groups was applied. The significance thresholds in Welch’s T-test were set to p<0.05, |FC|>3 and a mean signal of all samples >50^th^ percentile. Statistical and hierarchical clustering analyses (HCA) were done with the ArrayTrack Software [64] or MeV 4.8.0 of TM4 Microarray Software Suit [65]. In the tables and HCA the fold changes of the gene expression are given as Signal Logarithm Ratios (SLR) with the base 2.

Target genes for miRNAs were predicted with the software TargetScan by searching for the presence of conserved 8mer and 7mer sites that match the seed region of each miRNA [35]. A total of 152 target genes was predicted for the 8 regulated miRNAs**.**

### qRT-PCR Assays of Regulated miRNAs

Quantification of microRNA expression was carried out using TaqMan MicroRNA Assay kits according to the manufacturer’s protocol (Applied Biosystems, Foster City, CA). Prefabricated TaqMan MicroRNA Assays (containing microRNA-specific forward and reverse PCR primers and microRNA-specific Taqman MGB probe) were used to determine expression of miR-21 (ABI P/N 000397), miR-146b-5p (ABI P/N001097), miR-127 (ABI P/N000452), miR-433-3p (ABI P/N001028), miR-322 (ABI P/N001076), miR-184-3p (ABI P/N000485), miR-183 (ABI P/N002269), miR-96 (ABI P/N000186), miR-15a-5p (ABI P/N000389), miR-34a-5p (ABI P/N000426), miR-146a-5p (ABI P/N000468) and miR-182-5p (ABI P/N002599). Measurement of U6 small nuclear RNA (RNU6B) (ABI P/N 4373381) was used as housekeeping microRNA. Complementary DNA was generated using the Taqman MicroRNA Reverse Transcription (RT) Kit (ABI P/N 4366596) according to the manufacturer’s instructions. Reverse transcriptase reactions contained 10 ng of total RNA as the template, 5 µL of gene-specific stem-loop RT primer, 1.5 µL of 10 RT buffer, 0.15 µL of 100 mM dNTPs, 1 µL of MultiScribe reverse transcriptase, and 4.16 µL of nuclease-free water. The 15-µL reactions were incubated on a GeneAmp PCR System (Bio-Rad, Hercules, CA) for 30 minutes at 16°C, 30 minutes at 42°C, 5 minutes at 85°C and then held at 4°C. Quantitative RT-PCR was carried out using the BIORAD CFX384™ Real Time PCR Detection System. The 20-µL PCR reactions contained 1.33 µL of RT product, 10 µL of FastStart TaqManProbe Master (ROCHE P/N04673417001), 7.67 µL of nuclease–free water, and 1 µL of MicroRNA Assay (Applied Biosystem) buffer. Reactions were incubated at 95°C for 10 minutes, followed by 40 cycles of incubation at 95°C for 15 seconds and at 60°C for 1 minute. The threshold cycle data (CT) and baselines were determined using autosettings. The CT value was defined as the fractional cycle number at which the fluorescence passed the fixed threshold.

### MiRNA Target Prediction

The search for target genes of regulated miRNAs was carried out by use of two different web based tools, i.e. TargetScan, release 5.2 (http://www.targetscan.org/vert_50/). Here, the search was performed at default settings while the PITA searches (http://genie.weizmann.ac.il/pubs/mir07/index.html) were done at the 3′-UTR regions of target genes with a p<0.05 by defining the probability distribution of random matches with a minimum miRNA seed length of 7 nt.

### Clinical Significance of Predicted Gene Targets

The Human Protein Atlas project combines high-throughput generation of affinity-purified polyclonal antibodies with immunohistochemistry-based protein profiling on tissue and cell microarrays containing normal human tissue, cancer tissue and cell lines [66], [67]. At present, 11,200 unique proteins, corresponding to more than 56% of all human protein-encoding genes, have been analyzed. All protein expression data, including underlying high-resolution images, are publicly available at www.proteinatlas.org [66], [67]. Distinct and heterogeneous protein expression patterns were analyzed in the twelve cases of NSCLC available in the Human Protein Atlas.

### Gene Ontology Analysis

Out of the list of 152 predicted gene targets all repressed genes (n = 60, see Figure 6) were uploaded to the FatiGO [68] software. Then, a Fisher’s exact test was used to check for significant over-representation of GO terms in the submitted dataset against the remaining ones from the *Homo sapiens* genome.

## Supporting Information

Table S1 List of 152 human genes, predicted to be regulated by the 8 significantly regulated miRNAs identified in c-Raf transgenic mice. For each gene the table indicates the percentage of increase or decrease in protein expression as determined by immunohistochemistry using the signal intensity for lung tumor as compared to healthy surrounding tissue as reported in the Human Protein Atlas database. N/A = Non-Available data.
(XLS)
